# Integrated FDG-PET/CT imaging is useful in the approach to carcinoid tumors of the lung

**DOI:** 10.1186/1749-8090-8-223

**Published:** 2013-12-04

**Authors:** Alessandro Stefani, Antonella Franceschetto, Jessica Nesci, Beatrice Aramini, Chiara Proli, Shaniko Kaleci, Alessandra Casolo, Lucia Massi, Christian Casali, Uliano Morandi

**Affiliations:** 1 Department of Thoracic Surgery, University of Modena and Reggio Emilia, Modena, Italy; 2 Department of Nuclear Medicine, University of Modena and Reggio Emilia, Modena, Italy; 3 Department of Biostatistics, University of Modena and Reggio Emilia, Modena, Italy

**Keywords:** Bronchial carcinoid, Positron emission tomography, Standardized uptake value

## Abstract

**Background:**

Carcinoids enter the differential diagnosis of the solitary pulmonary nodule. Bronchial carcinoids have been traditionally considered as FDG-PET negative but recent studies have found an higher sensitivity of integrated FDG-PET/CT for the detection of these neoplasms. The purpose of this study was to investigate the value of integrated FDG-PET/CT for the evaluation of SPN suspected to be carcinoids.

**Methods:**

All patients with pathologically proven bronchial carcinoids who had FDG-PET/CT scans between 2006 and 2012 have been retrospectively reviewed. PET/CT was performed with the same scanner and the same technique for all patients. The following data were retrieved: age, sex CT findings (side, location, size, shape, margins), SUVmax, type of operation, pathological findings (size and number of mitoses). Regarding PET findings, only SUVmax was considered, whereas the visual assessment was not undertaken. Carcinoids were defined as typical and atypical and as central and peripheral. The long-term follow-up was also recorded. The SUVmax was compared with the other clinical, radiological and pathological variables to find any significant difference or correlation.

**Results:**

Twenty-five patients were retrieved, 24 typical and one atypical carcinoid, 21 peripheral and 4 central lesions. The mean diameter on CT-scan was 25.3 mm and the clinical size correlated well with the pathological size. Sixty percent of the tumors were ovoid and 68% had smooth margins. The mean SUVmax was 3.6 (range 1.4-12.9). All the lesions were completely resected. The regression analysis showed a direct correlation between the SUVmax and the tumor size (p = 0.004). No further correlations were found between the SUVmax and the other variables. None of the patients had recurrent disease or died during the follow-up.

**Conclusions:**

Our study showed that FDG-PET/CT might be a useful tool in the evaluation of SPNs suspected to be bronchial carcinoids. When a solitary pulmonary nodule shows an ovoid/round shape and smooth margins on the CT scan and demonstrates an FDG uptake higher than that of the normal lung and with a SUVmax value >1-1.5, a carcinoid should be suspected. If benign lesions can be presumably excluded, surgical resection or at least a biopsy of the lesion is recommended.

## Background

Bronchial carcinoids account for 1-2% of lung neoplasms [1]. They are histologically classified as typical (low-grade malignancies, accounting for 85-90% of all carcinoids) and atypical (intermediate-grade) [2]. Eighty percent of pulmonary carcinoids are endobronchial in origin and they can be easily diagnosed by fiberoptic bronchoscopy biopsy, whereas 20% occur peripherally and manifest as solitary nodules or masses on chest imaging, usually in asymptomatic patients [3]. These peripheral forms are part of the differential diagnosis of solitary pulmonary nodule (SPN). Imaging with somatostatin-receptor scintigraphy could be used in cases of a suspected carcinoid [4,5] but, when an SPN is found on a computed tomography (CT) scan, many diseases other than carcinoid can be suspected, such as lung carcinoma, hamartoma, inflammatory or infectious lesions. Thus, in clinical practice, somatostatin-receptor scintigraphy is not the examination of choice after CT in the evaluation of SPN. 18 F-fluorodeoxyglucose positron emission tomography (FDG-PET) is the preferred examination in this setting [6]. More recently, integrated FDG-PET/CT has been shown to be an effective tool to differentiate malignant from benign lesions [7]. But carcinoids, especially in the typical form, show a low metabolic activity which reduces the sensitivity of FDG-PET for the diagnosis of these neoplasms [8]. In fact, bronchial carcinoids have been traditionally considered FDG-PET negative and this understanding led many physicians to consider this technique as not a valuable tool for the diagnosis of these tumors. Conversely, recent studies have found a higher sensitivity of FDG-PET for the detection of carcinoids, for both the typical and atypical forms [9-11], although these studies have reported series with a limited number of patients. Therefore, peripheral carcinoids presenting as SPNs remain a diagnostic challenge.

The purpose of this study was to investigate the value of integrated FDG-PET/CT for the evaluation of bronchial carcinoids by reviewing our institutional experience since 2006.

## Methods

### Patients

We retrospectively reviewed all the patients with pathologically proven carcinoid tumors who had FDG-PET/CT scans at our institution, between January 2006 and December 2012.

The study was approved by the Institutional Review Board of the University of Modena and Reggio Emilia.

At our institution FDG-PET/CT scanning was introduced in 2006 and has been used in the preoperative diagnostic and staging work-up of patients with SPN suspected to be lung tumors. The following data were retrieved from the clinical records of patients affected by carcinoid who entered the study: age, sex, CT findings, FDG-PET/CT findings, preoperative diagnosis (if available), type of operation, number of mitoses and surgical stage. Central carcinoid was defined if the tumor was visible on the bronchoscopy examination, otherwise the tumor was defined as peripheral, regardeless of its location within the lung parenchyma. When the tumor was visible on bronchoscopy, an endoscopic biopsy of the lesion was undertaken.

Patients were referred to surgery because of the suspicion of a malignant lesion, predominantly based on CT and PET findings. Enhanced CT-scans were acquired with helical technique in all patients, before FDG-PET/CT examination. The indication to perform an FDG-PET/CT was based on the results of CT scan. All patients underwent surgical resection and the final diagnosis was based on the histopathology of the surgical specimens. Lateral muscle-sparing thoracotomy was the surgical approach. Surgical resection was lobectomy or limited resection in all cases and mediastinal lymph node dissection was routinely performed. A frozen section examination was undertaken in all the cases without preoperative diagnosis.

### FDG-PET/CT

All the FDG-PET/CT scans were reviewed by nuclear medicine experts, who were not blinded.

Whole-body FDG-PET/CT was performed with a combined PET/CT scanner (GE Discovery DSTE, General Electric medical systems, Milwaukee, WI, USA). This scanner allows multi-detector row helical CT scanning with 16 array of detectors. The technical parameters used for the CT were as follows: a detector row configuration of 4 sections of 3.75 mm thickness, pitch of 1.375:1, KVp: 120, a gantry rotation speed of 0.8 s and an amperage of 80–250 mA, modulated to maintain a noise index of 30, in *free breath*. The CT examination was used for attenuation correction of PET images.

The patients fasted for at least 6 hours prior to the scan. After verification of serum glucose levels of ≤ 150 mg/dl, the patients received an intravenous injection of 3.7 MBq/Kg of FDG. The scans were started 60 to 70 minutes after the injection and scanned the area from the base of the brain to the mid-thigh. The emission PET scan was obtained with a 2.5 min acquisition per bed position; typically, six or seven bed positions are obtained. The scanner operates in the three-dimensional mode and the images were reviewed in axial, coronal and sagittal formats. For this study, visual analysis was not performed and we considered only the tumor activity using the maximum standardized uptake value (SUVmax), to minimize the partial volume effect on the uptake value. The SUVmax is a semiquantitative measurement of tissue metabolic rate and represents the maximum measured activity at the region of interest (ROI), placed manually over the most intense area of FDG accumulation, on the axial attenuation corrected PET images.

CT images of both helical CT scan and integrated PET/CT were analyzed for the tumor location, tumor size, shape and margin characteristics, presence of calcifications, necrotic low-attenuation areas and associated post-obstructive atelectasis or pneumonia. The tumor location was expressed in terms of side and lobe and the tumor size was defined by the longest diameter. The tumor shape was classified as ovoid, round or lobulated and the tumor margins as smooth or spiculated.

### Pathology

Typical carcinoids (TC) and atypical carcinoids (AC) were defined on the basis of the current World Health Organization criteria as follows [12]: TC has <2 mitotic figures per 2 mm^2^ and no necrosis, whereas AC has 2 to 10 mitotic figures per 2 mm^2^ or evidence of necrosis. The presence of lymph node metastases was reported and expressed as N1 (hilar) or N2 (mediastinal).

### Survival

The patients were routinely followed up at our institution and the survival data were available in our clinical databases. Follow-up was updated on May 2013 for all patients.

### Statistical analysis

The objective of the study was to investigate the accuracy of FDG-PET/CT in the evaluation of bronchial carcinoids. The SUVmax of all the lesions was calculated and compared with the other clinical, radiological and pathological variables, to find any significant difference or correlation.

The statistical analysis was conducted using the SPSS® statistical software (Chicago, Ill, USA). Data were expressed in terms of frequencies, mean and standard deviation (SD). The comparisons were performed with the chi-square test for categorical variables and the t-test for continuous variables. The correlation between SUVmax and size was investigated using Spearman’s correlation coefficient and the goodness-of-fit measure of each linear model (R^2^) was reported. A p value of less than 0.05 was considered significant.

## Results

Twenty-five patients presenting with SPN, having FDG-PET/CT and undergoing subsequent surgical resection for suspected malignancy were found to have bronchial carcinoids.

The patients characteristics are presented in Table 1. There were 22 women (88%) and 3 men, and mean age was 58.8 years (range 29–78). No tumors showed features of ectopic hormonal secretion (carcinoid or Cushing syndrome). The left lung was involved in 14 cases (56%); the left upper lobe and right middle lobe were most frequently involved (9 and 8 cases, respectively). Most of the lesions were located peripherally (21/25, 84%). The mean diameter on the CT-scan was 25.3 mm (range 12–70, SD 14 mm), and most tumors were ovoid (n = 15, 60%) and had smooth margins (n = 17, 68%). Necrotic areas, calcifications, atelectasis or obstructive pneumonia were not found in any case.

**Table 1:** Clinical characteristics and PET/CT findings of the 25 patients

**Pts**	**age**	**sex**	**CT findings**					**PET findings**	**Surgical procedure**	**Histologic findings**	
			**Lobe location**	**Lesion location**	**Size (mm)**	**Shape**	**Margin**	**SUVmax**		**Cell type**	**No of mitoses**
1	78	F	LUL	Peripheral	12	ovoid	smooth	1.9	Lobectomy	Typical	1
2	62	F	LUL	Peripheral	18	ovoid	smooth	4.6	Lobectomy	Typical	1
3	64	F	RML	Peripheral	20	lobulated	smooth	12.9	Lobectomy	Typical	1
4	29	F	LLL	Central	70	ovoid	spiculated	9.7	Lobectomy	Typical	1
5	36	F	RML	Peripheral	28	lobulated	spiculated	4.2	Lobectomy	Typical	1
6	67	F	RML	peripheral	15	ovoid	spiculated	2.8	Lobectomy	Typical	1
7	74	F	LUL	Peripheral	18	ovoid	spiculated	2.4	Wedge resection	Typical	1
8	51	F	RML	Peripheral	15	ovoid	smooth	1.9	Lobectomy	Typical	1
9	49	F	RML	Peripheral	25	lobulated	smooth	6.0	Lobectomy	Typical	1
10	61	M	LUL	Peripheral	23	ovoid	smooth	1.5	Lobectomy	Typical	1
11	61	M	LUL	Central	42	ovoid	smooth	2.2	Lobectomy	Typical	1
12	51	F	LUL	Peripheral	23	lobulated	spiculated	3.0	Lobectomy	Typical	1
13	61	F	LLL	Peripheral	20	ovoid	smooth	4.5	Segmentectomy	Typical	0
14	35	F	LLL	Peripheral	38	lobulated	smooth	5.0	Lobectomy	Atypical	4
15	60	F	LLL	Central	55	round	spiculated	4.2	Lobectomy	Typical	1
16	72	F	RUL	Peripheral	24	round	smooth	3.1	Lobectomy	Typical	1
17	47	F	LLL	Central	40	ovoid	smooth	2.0	Lobectomy	Typical	1
18	63	F	RML	Peripheral	14	lobulated	smooth	2.4	Lobectomy	Typical	1
19	70	M	LUL	Peripheral	23	ovoid	spiculated	6.2	Wedge resection	Typical	0
20	61	F	RUL	Peripheral	13	lobulated	smooth	1.4	Lobectomy	Typical	0
21	60	F	RUL	Peripheral	15	ovoid	smooth	1.5	Wedge resection	Typical	1
22	60	F	RML	Peripheral	18	lobulated	smooth	1.8	Wedge resection	Typical	1
23	78	F	LUL	Peripheral	16	ovoid	smooth	1.8	Lobectomy	Typical	1
24	61	F	RML	Peripheral	34	ovoid	spiculated	3.1	Lobectomy	Typical	1
25	61	F	LUL	Peripheral	15	ovoid	smooth	1.7	Lobectomy	Typical	1

The mean SUVmax of the nodules was 3.6 and the median SUVmax was 2.8 (range 1.4-12.9, SD 2.7). The FDG-PET did not reveal significant uptake in hilar or mediastinal lymph nodes, and the CT scan was negative for suspected node metastases in all cases.

Tissue diagnosis was obtained by bronchoscopic biopsy, prior to surgical resection, in the 4 patients with central lesions (16%); in these cases a diagnosis of suspected carcinoid was achieved. In the other patients the diagnosis was obtained by frozen section examination and confirmed on histopathological analysis.

All the lesions were completely resected, in most cases by lobectomy (20/25, 80%). The final diagnoses revealed 24 TCs and one AC. The mean pathological size was 24 mm (range 7–68, SD 14.9) and the pathological dimensions were comparable with the clinical dimensions (p = 0.239). All the TCs showed one or no mitoses per 2 mm^2^, whereas the AC showed 4 mitoses per 2 mm^2^. The patient with AC had one metastatic hilar lymph node (N1) whereas the other cases were free of lymph node involvement.

**Figure 1:**
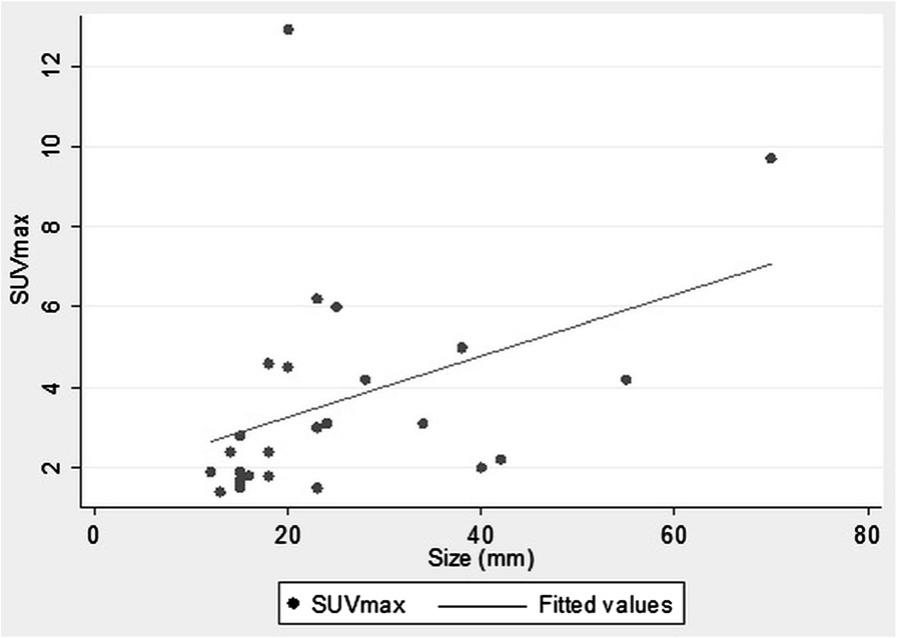
The linear correlation model between the SUVmax and tumor diameter shows a significant direct correlation.

All the variables were compared and the differences are as follows: regarding age and location, the patients with central lesions were younger than those with peripheral carcinoids (mean age 47 and 62 years respectively, p = 0.033); concerning side and location, all the central lesions were on the left side (p = 0.020); regarding location and size, the central lesions were larger than the peripheral ones (51.7 and 17.4 mm respectively, p < 0.01). The bivariate regression analysis showed a direct correlation between the SUVmax and the clinical tumor size: Spearman’s rho 0.552, R^2^ 0.30, p = 0,004 (Figure 1). Of the 11 tumors with a diameter <20 mm, only 2 (18%) showed a SUVmax >2.5 (2.8 and 4.6), and 50% of the tumors with a diameter >30 mm showed a SUVmax >2.5 (n = 3/6). A similar significant correlation was found when pathological size was considered in the regression analysis and compared to SUVmax. No further correlations were found between the SUVmax and the other clinical, radiological and pathological variables.

Follow-up was complete for all the patients, with a mean of 43 months (range 12–74 months). None of the patients had recurrent carcinoid disease or died during the follow-up.

## Discussion

Bronchial carcinoids enter the differential diagnosis of SPN or mass. On the CT scan they typically present as nodules or masses with an ovoid/round shape and smooth margins [3]. However, also hamartomas, metastases, infectious/inflammatory lesions or, in some cases, lung carcinoma, can present this radiological aspect. On the other hand, carcinoids can present with spiculated margins and irregular shape, although less commonly (8/25 patients, 32% in our series).

Because carcinoids express somatostatin receptors on their surface, functional imaging with radiolabeled somatostatin analogues is the examination of choice [11,13]. In clinical practice, however, when a SPN or mass is detected by CT scan, the diagnostic work-up is frequently completed using FDG-PET, or, preferably, integrated FDG-PET/CT, if available [14]. But pulmonary carcinoids have been traditionally described as tumors with low grade of FDG uptake, most likely because of their low metabolism and slow growth. Therefore, FDG-PET has been commonly understood to have a reduced sensitivity for carcinoids and a limited role in the diagnostic work-up of these lesions. This conviction originated from the first work on FDG-PET and carcinoids by Erasmus in 1998 [8], who reported on 7 carcinoids, of which 6 were hypometabolic (86%) and thus erroneously considered benign. Later studies, however, did not confirm those findings. Wartski described two carcinoids with intense FDG uptake (SUVmax 4.8 and 10.6) [15]. Kruger reported 13 carcinoids of which 54% were hypermetabolic (SUVmax >2.5) [16]. Daniels studied 16 patients and found a sensitivity for FDG-PET detection of carcinoids of 75% [9]. Chong found that the uptake in 3 of 7 carcinoids (43%) was higher than the mediastinal uptake and they were considered positive on the PET/CT scan [10]. Kayani reported that 9 of 13 patients with carcinoids (69%) showed significant FDG uptake and were considered to have malignant lesions [11]. Jindal found an overall detection rate by FDG-PET/CT of 70% in a series of 20 carcinoids [13].

The method of assessment of the nodule uptake is of utmost importance for an adequate evaluation of the accuracy of FDG-PET for carcinoids. The sensitivity of FDG-PET for carcinoids was generally investigated on the basis of a visual assessment or of a SUVmax cut-off. Regarding the visual assessment, the PET scan results were tipically interpreted as positive when the nodule activity was greater than the background mediastinal pool activity (8–10). When the SUVmax was used to define the PET positivity, a cut-off of 2.5 was commonly applied [11,13,16]. Both methods of assessment of nodule activity are derived from the evaluation of lung carcinoma [17]. But carcinoids, especially the typical forms, show a lower metabolic activity with respect to lung carcinoma, therefore, a different method should be applied for the evaluation of FDG uptake. We hypothesized that it may be more appropriate to compare the nodule activity with the normal lung and not with the mediastinal blood pool activity, so that the majority of the nodules can be easily visualized, as noted by Erasmus [8]. The evaluation of SUVmax is mandatory with this method of assessment. However, the cut-off value of 2.5 appears to be no longer adequate, especially for TCs. As reported by other authors [8,11,13,14], also in our series a number of TCs showed a SUVmax <2.5 (12 patients of 24, 50%). If a cut-off of 1.5 was fixed, no false-negative results would have been present in the series by Erasmus (0/6 patients) [8] and Kayani (0/11) [11] and reduced false-negative rates of 23%, 8% and 4% would have been found in the series by Jindal [13] and Kruger [16] and in our series, respectively.

**Figure 2:**
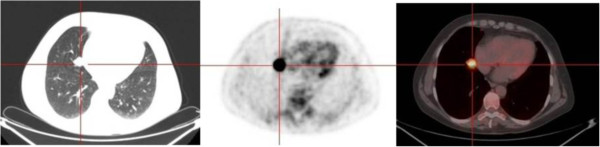
**The integrated FDG-PET/CT reveals a small TC (20 mm) demonstrating high uptake (SUVmax 12.9).** From left to right: CT, PET and fused image.

**Figure 3:**
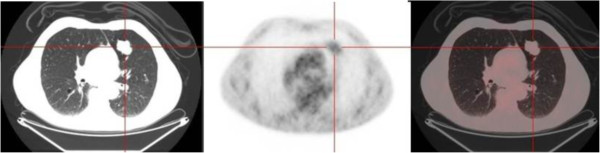
**The integrated FDG-PET/CT reveals a large TC (42 mm) demonstrating low uptake (SUVmax 2.2).** From left to right: CT, PET and fused image.

FDG uptake of carcinoids was found to be related to the histologic type, proliferation rate and size of the lesion. Several authors found that ACs showed a significantly higher FDG uptake than TC and in many cases this uptake was comparable to that of lung carcinoma [9-11,13,15]. This is because ACs show high proliferation rates compared to TCs. Our series included 24 TCs (96%) and one AC, thus an analysis based on the histologic subtype was not feasible. Regarding the tumor size, for lung carcinoma it is well known that the intensity of the FDG uptake can be directly correlated to the tumor dimensions [18,19]. Erasmus [8] and Kruger [16] demonstrated that the SUVmax was directly related to the tumor diameter also for bronchial carcinoids. Also Daniels reported the correlation between the FDG uptake and the size of the neoplasm but the SUVmax was not calculated in his work [9]. In our series we found a direct correlation between the SUVmax and the size of the tumor, when both the clinical and pathological diameters were investigated (Figure 1). This relationship may partly explain the variability in the FDG uptake in our series of TCs (SUVmax range 1.4 – 12.9), although some exceptions are present (Figures 2 and 3). We did not find any association between the SUVmax and other tumor features, such as location, shape or margins; similar associations have not been reported in the literature.

Some authors found a correlation between the staging of lung carcinoids by FDG-PET and surgical staging [9,10]. In our series, FDG-PET was negative for nodal metastases in all patients. This finding correlated well with the pathological findings, because all the patients resulted pN0, except for the AC patient, who had pN1 disease. However, because our series lack of a comparison group of N + patients, conclusions regarding PET utility for nodal disease remain uncertain.

In our study, 4 patients received a preoperative diagnosis of TC by fiberoptic bronchoscopy. It is generally accepted that FDG-PET is not mandatory in the clinical evaluation of patients with biopsy-proven pulmonary carcinoid [9,11] and we agree with this statement. Our series includes patients with biopsy-proven TC only because they underwent PET/CT before bronchoscopy during their staging work-up. The disproportionate number of peripheral carcinoids in our study population reflects the clinical policy to consider that PET/CT is not necessary in cases of a preoperative diagnosis of bronchial carcinoid, which is usually obtained in central forms by bronchoscopy.

Long-term prognosis for patients affected by TC is favourable, with 5-year survival rates higher than 95% after radical surgical resection [20]. This rate is consistent with our findings, which showed no deaths or recurrences of carcinoid disease at follow-up.

In our study, all the lesions were clearly detectable as SPNs on helical CT. In many cases, ovoid/round shape and regular margins suggested a carcinoid, and the main differential diagnosis was with hamartoma or other benign lesions. In a minority of cases, spiculated margins and irregular shape led to inclusion of lung carcinoma in the differential diagnosis. All of these lesions showed FDG uptake, therefore none was considered typically benign, and we proceeded to surgical resection in all cases. For the lesions with a lower uptake, a diagnosis of hamartoma was considered unlikely, because the FDG uptake is exceptional for hamartomas [21,22]; infections or inflammatory lesions remained in the differential diagnosis but they were considered less probable than carcinoids after a careful examination of the patient. Regarding the lesions with a higher uptake, a diagnosis of lung carcinoma, particularly a low-grade adenocarcinoma, was taken into account; in these latter cases, a further unconfirmed suspicion of carcinoid would not have affected the adequacy of the surgical indication.

The assessment of the nodule characteristic was performed analysing the images of enhanced helical CT-scan rather than CT images of the integrated PET/CT scan. The acquisition parameters of CT scan of the integrated PET/CT are not adequate for a careful assessment of the nodule features, especially for small lesions. Moreover, the enhanced helical CT scan is usually the first approach to the SPN following the chest-x-ray and, therefore, is available for all patients.

The main limitation of this study is its retrospective nature. The data only included nodules that were resected and may underestimate the number of PET false-negative lesions.

## Conclusions

We believe that for cases in which an SPN with an ovoid/round shape and smooth margins on the CT scan reveals an FDG uptake higher than that of the normal lung and with a SUVmax value >1-1.5, a bronchial carcinoid should be considered and surgical resection or at least a biopsy of the lesion should be recommended.

Further studies comparing the PET/CT findings of carcinoids, hamartomas, low-grade adenocarcinomas and infectious/inflammatory lesions should be advocated.

## Abbreviations

18F-FDG-PET: 18F-fluorodeoxyglucose positron emission tomography; AC: Atypical carcinoids; CT: Computed tomography; ROI: Region of interest; SD: Standard deviation; SPN: Solitary pulmonary nodule; SUV: Standardized uptake value; TC: Typical carcinoids.

## Competing interests

The authors declare that they have no competing interests.

## Authors’ contribution

AS conceived of the study, participated in its design and drafted the manuscript. AF, AC and LM performed the SUVmax analysis, helped to draft the manuscript and drafted Figures 2 and 3. JN and CP retrieved the data and created the database. BA retrieved the data and performed the language revision. SK performed the statistical analysis and drafted Figure 1. UM participated in conceiving the study and its design and coordinated the study. All authors read and approved the final manuscript.
